# Chronic fatigue syndrome in an ethnically diverse population: the influence of psychosocial adversity and physical inactivity

**DOI:** 10.1186/1741-7015-9-26

**Published:** 2011-03-21

**Authors:** Kamaldeep S Bhui, Sokratis Dinos, Deborah Ashby, James Nazroo, Simon Wessely, Peter D White

**Affiliations:** 1 Centre for Psychiatry, Wolson Institute of Preventive Medicine, Barts and the London School of Medicine and Dentistry, Queen Mary University of London, London, UK; 2 Division of Epidemiology, Public Health and Primary Care, Imperial College London, UK; 3 School of Social Sciences, University of Manchester, Manchester, UK; 4 Department of Psychological Medicine and Psychiatry, Institute of Psychiatry, King's College London, London, UK

## Abstract

**Background:**

Chronic fatigue syndrome (CFS) is a complex multifactorial disorder. This paper reports the prevalence of chronic fatigue (CF) and CFS in an ethnically diverse population sample and tests whether prevalence varies by social adversity, social support, physical inactivity, anxiety and depression.

**Methods:**

Analysis of survey data linking the Health Survey for England (1998 and 1999) and the Ethnic Minority Psychiatric Illness Rates in the Community (EMPIRIC) study undertaken in 2000. The study population comprised a national population sample of 4,281 people ages 16 to 74 years. CF and CFS were operationally defined on the basis of an interview in the EMPIRIC study, alongside questions about psychosocial risk factors. Previous illnesses were reported in the Health Survey for England during 1998 and 1999, as was physical inactivity.

**Results:**

All ethnic minority groups had a higher prevalence of CFS than the White group. The lowest prevalence was 0.8% in the White group, and it was highest at 3.5% in the Pakistani group (odds ratio (OR), 4.1; 95% confidence interval (95% CI), 1.6 to 10.4). Anxiety (OR, 1.8; 95% CI, 1.4 to 2.2), depression (OR, 1.4; 95% CI, 1.1 to 1.8), physical inactivity (OR, 2.0; 95% CI, 1.1 to 3.8), social strain (OR, 1.24; 95% CI, 1.04 to 1.48) and negative aspects of social support (OR, 2.12; 95% CI, 1.4 to 3.3) were independent risk factors for CFS in the overall sample. Together these risk factors explained ethnic differences in the prevalence of CFS, but no single risk factor could explain a higher prevalence in all ethnic groups.

**Conclusions:**

The prevalence of CFS, but not CF, varies by ethnic group. Anxiety, depression, physical inactivity, social strain and negative aspects of social support together accounted for prevalence differences of CFS in the overall sample.

## Background

Chronic fatigue syndrome (CFS), sometimes also called myalgic encephalomyelitis, is a debilitating condition characterised by unexplained fatigue that lasts for at least 6 months alongside other symptoms that are required for a diagnosis of CFS: headaches, unrefreshing sleep, muscle pain and memory and concentration problems [1]. The prevalence is between 400 and 2,500 adults per 100,000 population [1,2]. Chronic fatigue (CF) alone, without meeting the full criteria for CFS, is more prevalent but less disabling than CFS [3]. Although the exact pathogenesis of CFS is unknown, research implicates infection, endocrine dysfunction, autonomic nervous system imbalance, depressed mood and altered immunity [1,2]. Psychosocial factors and physical inactivity have been proposed to be of aetiological significance [1,2], but there is little research on the relative importance of physical inactivity and psychosocial factors in population samples. Cultural factors are known to influence psychosocial risks for many health conditions; therefore, studies in ethnically diverse populations may yield more information about the relative importance of sociocultural, psychological and behavioural risk factors.

Early reports of CFS from clinic populations seemed to suggest that CFS was more common in women, White majority population and the middle classes [4,5]. In contrast, some recent population-based research in the United States and the United Kingdom shows that the prevalence of CFS, like many illnesses [6] is actually higher among people of lower socioeconomic status and minority cultural or ethnic groups [3,7-11]. Psychosocial influences include social support, which is a protective factor against CFS [12-14], whereas social strain, including gender disadvantage and financial strain, are known risk factors for poor health in general and for CFS in particular [7,15]. Cultural factors include work-related discrimination, assaults and insults; these are more common amongst some ethnic minorities and are important risk factors for a number of health conditions [16-18]. These stressors have not been investigated in CFS.

Although physical illness may potentially explain greater reports of fatigue [19], physical illnesses that cause fatigue are among the exclusion criteria for a diagnosis of CFS [1,2]. For example, the latest international criteria (Centers for Disease Control (CDC), 2006) [20] allow a CFS diagnosis in the presence of long-standing physical illnesses only if these are stable, treated and do not account for fatigue (for example, hypothyroidism, diabetes mellitus and cancer). In contrast, depression and anxiety can cause fatigue and are common in patients with CFS, but are allowable in meeting the CDC criteria for CFS [2,21]. Therefore, ethnic variations in the prevalence of anxiety and depression may explain variations of CFS prevalence.

Some studies suggest that physical activity is an effective intervention for CFS [6]. However, the role of physical inactivity and overactivity in causing CFS is uncertain [2,22-24]. Physical inactivity may play a role in maintaining CFS [25] and is known to be more common among some cultural and ethnic groups. For example, Indian, Pakistani, Bangladeshi and Chinese men and women were the least likely, in a health survey conducted in England, to be as active as recommended in health guidelines [26,27]. Therefore, varying levels of physical activity may explain variations in CFS.

### Objectives

This paper presents the findings of a Medical Research Council (UK)-funded study to estimate the population prevalence of CF and CFS in an ethnically diverse sample. In this study, we tested whether there is a consistently higher prevalence of CF and CFS in specific cultural and ethnic groups and whether variations in prevalence can be explained by social adversity (social strain and perceived discrimination), social support, physical inactivity, anxiety and depression.

Our hypothesis was as follows: (1) CF and CFS show differing patterns of prevalence across ethnic groups, and this difference is independent of variations by age, sex and socioeconomic status; and (2) prevalence variations may be explained by variations in psychosocial risk factors and physical inactivity, such that (a) social adversity such as social strain, low social support and perceived discrimination account for a higher prevalence of CFS; (b) physical inactivity accounts for a higher prevalence of CFS; and (c) anxiety and depression account for a higher prevalence of CFS.

## Methods

Ethical approval for data collection was obtained from the North Thames Multi-Centre Research Ethics Committee and ratified by all Local Research Ethics Committees in England.

### The study population

**Figure 1:**
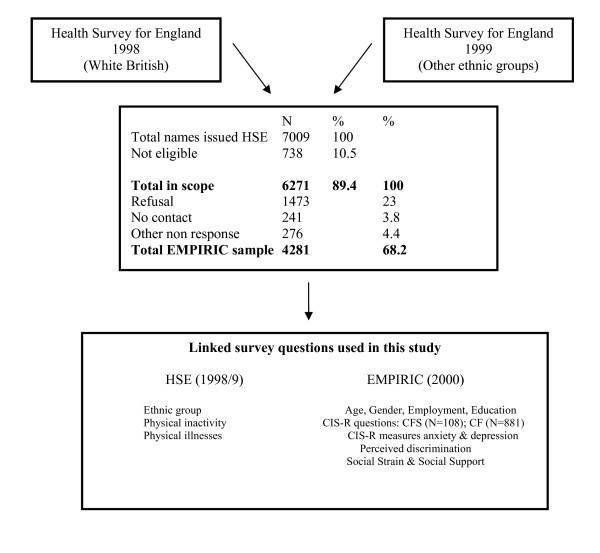
**Flow chart of sampling for the Ethnic Minority Psychiatric Illness Rates in the Community study**.

The Health Survey for England (HSE) comprises a series of annual surveys beginning in 1991. It was designed to provide regular information on the nation's health. The detailed methods used in the Ethnic Minority Psychiatric Illness Rates in the Community (EMPIRIC) study to sample from HSE have been published [27-29]. Therefore, in this paper, we briefly set out the sample methods. The EMPIRIC national population survey took place in 2000 and sampled White British participants from the 1998 Health Survey for England (HSE98) [26]. The 1999 Health Survey for England (HSE99) (the ninth in the series) was the first to increase the representation of ethnic minority groups, boosting the sample of adults from Black Caribbean, Indian, Pakistani, Bangladeshi and Irish communities. So, for the EMPIRIC survey, people from these ethnic groups were sampled starting from HSE99 [27] (see Figure 1). The EMPIRIC survey of 4,281 people ages 16 to 74 years included White British (*n *= 837), White Irish (*n *= 733), Black Caribbean (*n *= 694), Bangladeshi (*n *= 650), Indian (*n *= 643) and Pakistani (*n *= 724) people [27,28]. Response rates were highest among the White (71%) and Irish (72%) groups and lowest among the Indian group (62%). Weighting in EMPIRIC analyses ensured that the sample was representative of the population samples in the HSE annual surveys by removing different probabilities of selection. The EMPIRIC study included structured and validated questions about demographic variables and measures of social adversity and measures of anxiety and depression [29].

### Measuring CF and CFS

A question asking about fatigue in the Revised Clinical Interview Schedule (CIS-R) has previously been used to operationally define CF [7] and to validate more extensive measures of CF and CFS [3,30] (see Appendix). Therefore, to define CF and CFS, we used the fatigue question and other stem questions from the CIS-R (these questions were distinct from measures of anxiety and depression symptoms). The questions asked about the following symptoms: (1) getting tired and lacking energy and whether there were any reasons for this (such as physical illness), (2) having any problems with concentrating or noticing any problems with forgetting things, (3) having problems with falling asleep or with getting back to sleep, (4) any sort of ache or pain or being troubled by any sort of discomfort such as headache or indigestion.

Participants also rated the duration of symptoms. Using these items, we defined CF as the presence of unexplained fatigue for a period of 6 months. We defined a CFS-like syndrome as closely as possible to the international criteria for CFS [21]. Although the literature does at times make a distinction between CFS-like syndrome and CFS, this is not consistently followed, for example, the published studies on which the methods in this paper were adopted [3,7,31]. Therefore, in this paper, we make explicit the study methods and that the operational definition of CFS is compatible with what has been called by some a CFS-like syndrome. We counted as CFS those individuals with all four of the above symptoms, each lasting for at least 6 months. Our definition of at least four symptoms meets the threshold criteria, as CFS is usually diagnosed on the basis of four of eight symptoms, including fatigue [21]. Sensitivity analyses showed that when a fatigue syndrome was defined using fatigue and only two additional symptoms rather than three, the findings from the analyses were identical to the findings for CF. This supported the notion that our definition of CFS did identify a group that was distinct from the CF group.

The EMPIRIC study (2000) [28,29] was undertaken by following up and oversampling people from HSE98 and HSE99 [26,27]. We obtained the individual identifier codes that linked the two data sets, which permitted us to search for physical illnesses reported in HSE98 and HSE99. We were able to identify diseases that might exclude a diagnosis of CFS (31 of 108 (28.7%) participants met the criteria for CFS): cancer, diabetes, epilepsy, arthritis or fibrositis and infectious or parasitic disease. However, we did not know the duration of these physical diseases, whether they were in remission or whether they were causing the fatigue at the time of the HSE survey. Although one analytic approach might be to exclude all those individuals with these physical illnesses, given the lack of information about duration, degree of fatigue and whether it was being treated, this approach might introduce a significant selection bias. We therefore included those participants with the specified conditions in our sample and adjusted for all these illnesses in all of our logistic regression analyses where CFS was an outcome. This adjustment was part of the operational definition; therefore, we do not show the unadjusted analyses. This approach provided estimates of risk independent of physical illnesses that might cause fatigue. This method was supported by sensitivity analyses, which showed that the odds ratios following our analyses did not differ whether we included or excluded people with these physical illnesses. Therefore, using these operational definitions of CF and CFS, linked closely to carefully defined analytic plans, we tested the hypotheses by analysis of data collected in the EMPIRIC study [29].

### Measuring common mental disorder

The EMPIRIC study included the CIS-R, which asks about 14 symptoms in the week preceding a structured interview [32]. Anxiety and depression subscales of the CIS-R were used in the analyses (both are scored on a scale from 0 to 4). These were distinct from the questions about fatigue. The CIS-R shows good face content and construct validity in culturally diverse populations [28,29,32].

### Social adversity

Social class was classified by the head of household's occupation recoded into a 'manual' or 'nonmanual' group. Other measures of socioeconomic position were educational qualifications (recoded into no qualifications, degree or secondary school qualifications, or foreign qualifications) and employment status (employed or unemployed, retired or other economically inactive status). Social support was measured by the Close Persons Questionnaire [31]. Scores were classified using usual validated thresholds into 'low', 'medium' or 'high' levels of confiding or emotional support (seven items), practical support (four items) and negative aspects of close relationships (four items) [31].

Chronic social strain was measured by questions from the Whitehall II study [15,33]. The five-point Likert scale responses for each domain were dichotomized: (1) problems with relatives and problems with finances (always, often, sometimes and seldom or never) and (2) problems with essentials, problems with housing and problems with neighbourhood (few or no problems and many or great problems) [33]. These were summed to give a total score (on a scale from 0 to 4), where a higher score indicated more frequent (for relatives and finances) or greater social strain. This total score (from 0 to 4) was recoded into tertiles.

Perceived discrimination questions asked about insults in the preceding 12 months; unfair treatment at work regarding promotions; and refusals of employment; and assaults in relation to race, religion or ethnic group. Responses of 'Yes' or 'No' (score of 1 or 0, respectively) were summed to produce a total discrimination score from 0 to 4. These questions have been used in previous studies of perceived discrimination and health [34].

### Physical inactivity

Physical activity levels were measured in the HSE98 and HSE99, so they preceded the measures of CF and CFS in the EMPIRIC study (measured in 2000). The intensity of physical activity was assessed by an aggregate measure of the physical demands of people's jobs and four other types of activity: housework, gardening and 'do-it-yourself' jobs around the house (including painting, decorating, repairs), walking, sports participation and exercise. The 'intensity level' of activity was already derived in the original HSE data from the estimated energy costs of each activity: vigorous, moderate, light and inactive [26,27]. These were recoded into two groups: vigorous or moderate activity versus no activity or light activity. The number of days in the preceding 4 weeks during which subjects had participated in at least a moderate level of activity for at least 30 minutes daily was the recommended level of healthy activity at the time of the original HSE surveys [35,36]. The total number of active days that each informant reported was divided by four to produce an (average) weekly figure of <1 day, 1 to 4 days or 5 or more days weekly, so that the policy and clinical implications for recommended activity might more easily be discerned as activity in an average week. For each activity variable, the highest activity levels (intensity and frequency) were used as the reference group.

### Questionnaire and measures

The EMPIRIC study piloted and then used a structured questionnaire that was available in five languages (Hindi, Gujarati, Punjabi, Urdu and Bengali) [27]. A letter introduced the study before the survey. Gender and language matching were used whenever requested.

### Statistical analysis

All analyses used the survey commands in Stata version 10 software (StataCorp, College Station, TX, USA) and were weighted for probability sampling and nonresponse (in HSE99 and EMPIRIC) using established methods with these data to represent population estimates [27,29]. Unweighted descriptive data are presented, but all regression models were weighted. Differences between weighted means were assessed using weighted regression, with an overall *P *value reported. Logistic regression models were built using likelihood ratio (LR) tests to justify inclusion and then retention of variables in the models. Gender did not appear to contribute significantly to the models, but, given its importance, it was retained in all regression models.

The unadjusted associations of demographic factors, including ethnic groups, with CF and CFS are presented in Table 1. Further analyses then investigated univariate associations of social strain, perceived discrimination, social support, physical inactivity and anxiety and depression variables with CFS (Table 2). Table 3 sets out the relationships between these variables and ethnic groups.

**Table 1:** Demographic characteristics as correlates of CFS and CF (univariate)^a^

	CF		CFS	
	
Demographics	Percentage (*n*/*N*), unweighted	OR (95% CI), weighted	Percentage (*n*/*N*), unweighted	OR (95% CI), weighted
Ethnic group				
White	22.6 (189/836)	1	0.8 (7/835)	1
Irish	21.7 (159/732)	1 (0.7 to 1.3)	2.0 (15/732)	2.6 (0.98 to 6.9)
Black Caribbean	19.8 (137/691)	0.8 (0.6 to 1.01)	2.5 (17/690)	2.6 (1.04 to 6.7)^b^
Bangladeshi	19.4 (126/650)	0.8 (0.6 to 1.03)	3.4 (22/650)	3.2 (1.3 to 7.8)^b^
Indian	19.3 (124/643)	0.8 (0.6 to 1.1)	3.1 (20/643)	3.3 (1.3 to 8.3)^c^
Pakistani	20.2 (146/724)	0.8 (0.6 to 1.04)	3.7 (27/723)	4.3 (1.7 to 10.5)^d^
Sex				
Male	19 (368/1,939)	1	2.6 (50/1,937)	1
Female	22 (513/2,337)	1.2 (1.01 to 1.4)^b^	2.5 (58/2,336)	0.9 (0.6 to 1.4)
Age group, yr				
16 to 34	12 (198/1,646)	1	0.7 (12/1,645)	1
35 to 54	24.2 (416/1,720)	2.5 (2 to 3)^d^	3.5 (60/1,718)	5.4 (2.5 to 11.4)^d^
55 to 74	29.3 (267/910)	3.3 (2.7 to 4.2)^d^	4.0 (36/910)	5.5 (2.5 to 12.1)^d^
Marital status				
Married	22.4 (617/2,752)	1	2.9 (80/2,751)	1
Divorced/separated	31.1 (97/312)	1.5 (1.5 to 1.9)^c^	3.8 (12/312)	1.2 (0.6 to 2.5)
Widowed	28.5 (45/158)	1.2 (0.8 to 1.8)	3.8 (6/158)	1.4 (0.6 to 3.7)
Single	11.6 (122/1,054)	0.5 (0.4 to 0.6)^d^	1 (10/1,052)	0.4 (0.2 to 0.9)^b^
Employment status				
Employed	17.5 (397/2,272)	1	1.5 (35/2,271)	1
Unemployed	17.9 (30/168)	0.9 (0.6 to 1.5)	2.4 (4/168)	0.9 (0.3 to 3.5)
Retired	30.6 (144/470)	1.6 (1.2 to 2.1)^d^	3.4 (16/470)	1.7 (0.8 to 3.4)
Other economically inactive	23.4 (290/1,238)	1.2 (1.1 to 1.5)^b^	4.0 (49/1,236)	2.1 (1.3 to 3.4)^c^
Educational level				
Degree or above	19.2 (188/977)	1	1.4 (14/976)	1
A level/GCSE^e^	17.9 (275/1,536)	0.9 (0.7 to 1.1)	2.0 (30/1,534)	1.5 (0.7 to 3.2)
Foreign qualification	23.3 (38/163)	1.2 (0.8 to 1.9)	2.5 (4/163)	2.3 (0.6 to 8.3)
No qualification	25.5 (375/1,468)	1.4 (1.1 to 1.7)^c^	4.0 (59/1,468)	2.8 (1.4 to 5.7)^c^
Type of employment (head of household)				
Nonmanual labour	19.7 (334/1,693)	1	1.7 (29/1,692)	1
Manual labour	20.8 (484/2,326)	1 (0.8 to 1.2)	2.9 (68/2324)	1.6 (0.99 to 2.6)^b^
Place of birth				
United Kingdom	16.1 (238/1,476)	1	1.1 (16/1,475)	1
Other	23 (459/1,993)	1.3 (1.1 to 1.6)^c^	4.2 (84/1,992)	3.5 (1.8 to 6.8)^d^
Age of migration, yr				
<11	16.7 (391/2,335)	1	1.2 (28/2,333)	1
≥11	21.6 (223/1,031)	1.3 (1.1 to 1.6)^c^	4.3 (44/1,030)	4.2 (2.4 to 7.4)^d^

^a^CFS, chronic fatigue syndrome; CF, chronic fatigue; OR, odds ratio; 95% CI, 95% confidence interval; ^b^*P *≤ 0.05; ^c^*P *≤ 0.01; ^d^*P *≤ 0.001.

^e ^A Level = Advanced Level/GCSE = General Certificate of Secondary Education

**Table 2:** Social, health and physical inactivity correlates of CFS (univariate)^a^

CFS correlate	Percentage (*n*/*N*), unweighted	OR (95% CI), weighted
Social support		
Confiding/emotional support		
Low	3.1 (35/1,132)	1
Average	2.3 (38/1,675)	0.7 (0.4 to 1.3)
High	2.4 (33/1,398)	0.8 (0.5 to 1.5)
Practical support		
Low	1.9 (16/861)	1
Average	2.1 (28/1,317)	0.99 (0.5 to 2)
High	3.1 (62/2,032)	1.4 (0.7 to 2.7)
Negative aspects of support		
Low	0.6 (4/631)	1
Average	2 (30/1,472)	2.3 (0.7 to 7.6)
High	3.4 (72/2,103)	4 (1.2 to 12.4)^b^
Chronic strains		
Tertiles of total score		
Low	0.9 (9/1,023)	1
Average	2.2 (46/2,124)	2.3 (1.04 to 5)^b^
High	4.8 (53/1,100)	4.9 (2.2 to 10.6)^c^
Perceived discrimination		
Total score (means)	-	1.5 (1.1 to 2)^d^
Mental health (CIS-R subscales)		
Depression score	-	2.2 (1.9 to 2.6)^c^
Anxiety score	-	2.2 (1.9 to 2.6)^c^
Physical inactivity		
Days of moderate activity of ≥30 minutes/day in past 4 weeks^b^		
5 or more (high)	1.4 (16/1,126)	1
1 to 4 (medium)	1.3 (16/1,256)	1.02 (0.4 to 2.3)
1 or <1 (low)	4 (75/1,883)	3.3 (1.7 to 6.2)
Activity intensity		
Moderate/vigorous	1.7 (55/3,217)	1
Inactive/light	5.7 (53/926)	4.6 (2.9 to 7.2)

^a^CFS, chronic fatigue syndrome; OR, odds ratio; 95% CI, 95% confidence interval; CIS-R, Revised Clinical Interview Schedule; ^b^*P *≤ 0.05; ^c^*P *≤ 0.001; ^d^*P *≤ 0.01.

**Table 3:** Demographics, social, illness and physical activity characteristics of survey population by ethnicity^a^

	Sample size, *n *(%)							
	
Demographic variable	Total, *N *(%)	White	Irish	Black Caribbean	Bangladeshi	Indian	Pakistani	*P *value
		835 (19.5)	733 (17.1)	691 (16.1)	650 (15.2)	648 (15.1)	724 (16.9)	
Sex								
Male	1,950 (45.5)	365 (43.7)	323 (44.1)	280 (40.5)	321 (49.4)	316 (48.8)	345 (47.7)	≤0.04
Female	2,332 (54.5)	471 (56.1)	410 (55.9)	412 (59.5)	329 (50.6)	331 (51.2)	379 (52.3)	
Age, yr								
16 to 34	1,774 (41.4)	260 (31.1)	209 (28.5)	269 (38.9)	376 (57.8)	248 (38.3)	412 (56.9)	≤0.001
35 to 54	1,647 (38.5)	355 (42.5)	345 (47.1)	251 (36.3)	187 (28.7)	280 (43.2)	229 (21.6)	
55 to 74	862 (20.1)	221 (26.4)	179 (24.4)	171 (24.7)	88 (13.5)	120 (18.5)	83 (11.5)	
Marital status								
Married	2,674 (62.5)	518 (62.1)	461 (62.8)	279 (40.4)	446 (68.7)	464 (71.7)	506 (69.9)	≤0.001
Divorced/separated	313 (7.3)	75 (9)	78 (10.6)	84 (12.2)	18 (2.8)	32 (4.9)	26 (3.6)	
Widowed	142 (3.3)	36 (4.3)	25 (3.4)	18 (2.6)	28 (4.3)	19 (2.9)	16 (2.2)	
Single	1,150 (26.9)	205 (24.6)	170 (23.2)	310 (44.9)	157 (24.2)	132 (20.4)	176 (24.3)	
Employment status								
Employed	2,273 (55)	536 (66.4)	490 (69)	376 (57.4)	181 (29.1)	394 (61.9)	296 (42.4)	≤0.001
Unemployed	168 (4.1)	10 (1.2)	12 (1.7)	39 (6)	40 (6.4)	33 (5.2)	34 (4.9)	
Retired	446 (10.8)	130 (16.1)	96 (13.5)	97 (14.8)	33 (5.3)	49 (7.7)	41 (5.9)	
Other economically inactive	1,242 (30.1)	131 (16.2)	112 (15.8)	143 (21.8)	369 (59.2)	160 (25.2)	327 (46.8)	
Educational level								
Degree or above	951 (23.2)	213 (26.5)	195 (27.6)	172 (26)	62 (10.1)	187 (29.9)	122 (17.8)	≤0.001
A level/GCSE	1,549 (37.8)	356 (44.3)	294 (41.6)	272 (41.1)	172 (27.9)	221 (35.3)	234 (34.1)	
Foreign qualification	172 (4.2)	32 (4)	35 (5)	30 (4.5)	17 (2.8)	29 (4.6)	29 (4.2)	
No qualification	1,428 (34.8)	202 (25.2)	183 (25.9)	188 (28.4)	365 (59.3)	189 (30.2)	301 (43.9)	
Social class (employment type)								
Nonmanual labour	1,681 (41.9)	463 (56.3)	351 (48.5)	279 (42.7)	92 (16.4)	279 (44.6)	217 (34.5)	≤0.001
Manual labour	2,335 (58.1)	360 (43.7)	372 (51.5)	375 (57.3)	469 (83.6)	347 (55.4)	412 (65.5)	
Social support scores (tertiles)								
Confiding/emotional support								≤0.001
Low	1,137 (27)	225 (27.1)	194 (26.8)	226 (33.5)	154 (24)	168 (26.5)	170 (24.1)	
Average	1,667 (39.6)	301 (36.3)	244 (33.7)	239 (35.4)	313 (48.8)	262 (41.4)	308 (43.7)	
High	1,404 (33.4)	303 (36.6)	287 (39.6)	210 (31.1)	174 (27.1)	203 (32.1)	227 (32.2)	
Practical support								≤0.001
Low	892 (21.2)	200 (24.1)	149 (20.5)	179 (26.5)	68 (10.6)	145 (22.9)	151 (21.4)	
Average	1,308 (31)	296 (35.7)	259 (35.7)	253 (37.4)	130 (20.3)	185 (29.2)	185 (26.2)	
High	2,013 (47.8)	334 (40.2)	318 (43.8)	244 (36.1)	443 (69.1)	303 (47.9)	371 (52.5)	
Negative aspects of support								≤0.001
Low	646 (15.4)	159 (19.2)	113 (15.6)	135 (20)	31 (4.9)	100 (15.8)	108 (15.3)	
Average	1,484 (35.3)	365 (44)	331 (45.6)	225 (33.3)	126 (19.7)	200 (31.6)	237 (33.6)	
High	2,078 (49.4)	306 (36.9)	282 (38.8)	316 (46.7)	481 (75.4)	332 (52.5)	361 (51.1)	
Chronic strain mean scores^b^	1.62	1.34	1.44	1.71	2.31	1.38	1.53	≤0.0001
Perceived discrimination mean scores^b^	0.30	0.13	0.15	0.65	0.19	0.39	0.35	≤0.0001
Days of moderate activity ≥30 minutes/day in past 4 weeks								≤0.001
5 or more (high)	1,131 (26.5)	268 (32.1)	231 (31.6)	228 (33.1)	93 (14.3)	170 (26.2)	141 (19.5)	
1 to 4 (medium)	1,274 (29.8)	278 (33.3)	247 (33.7)	201 (29.2)	139 (21.4)	192 (29.6)	217 (30.1)	
1 or <1 (low)	1,870 (43.7)	288 (34.5)	254 (34.7)	260 (37.7)	418 (64.3)	286 (44.1)	364 (50.4)	
Activity intensity								≤0.001
Inactive/light	903 (22)	6 (0.7)	102 (14.4)	116 (17.4)	308 (49.9)	156 (24.9)	215 (31.3)	
Moderate/vigorous	3,201 (78)	795 (99.3)	606 (85.6)	549 (82.6)	309 (50.1)	470 (75.1)	472 (68.7)	
Anxiety mean scores^b^	0.30	0.31	0.38	0.29	0.20	0.29	0.32	0.005
Depressive mean scores^b^	0.43	0.41	0.41	0.42	0.36	0.46	0.51	0.003

^a^GCSE: General Certificate of Secondary Education. ^b^weighted mean

Table 4 sets out the nonparametric correlations between demographic and risk factors entered into the final regression model. Table 5 reports manual stepped logistic regression modelling. Explanatory variables were recoded to reduce the number of levels per variable. LR tests were used to justify addition and retention of each of the explanatory variables when added to the basic model. Using this manual stepped approach, we were able to investigate which specific risk factors, when added to the model, led to a reduction in risk in specific ethnic groups or all ethnic groups. The basic model included CFS as an outcome, as well as age (in years), socioeconomic position (measured by education, employment type and whether employed) and ethnicity. We entered social strain, perceived discrimination and social support first; only negative aspects of social support contributed to the model and were retained. Then, in a separate model, we entered physical activity intensity and frequency. The frequency of physical activity did not contribute significantly to the model and was excluded. Starting with the basic model again, we then added anxiety and depression scores (see Table 5). A full model showing independent effects of all of these variables is shown in Table 5.

**Table 4:** Relationship between independent variables with each other (Spearman's ρ)

Variable	Sex	Age	Education	Employment	Social strain	Discrimination	Negative social support	Physical inactivity	Depression	Anxiety
Sex	1.000									
Age	-0.0400^a^									
Education	-0.0381^a^	-0.2628^d^	1.0000							
Employment	0.2070 ^d^	0.1481^d^	-0.3573^d^	1.0000						
Social strain	0.0674^d^	-0.1135^d^	-0.0777^d^	0.1089^d^	1.0000					
Discrimination	-0.1176^d^	-0.0397^b^	0.1288^d^	-0.1038^d^	0.1293^d^	1.0000				
Negative social support	0.0445^b^	-0.1189^d^	-0.0443^b^	0.0753^d^	0.2077^d^	0.0467^b^	1.0000			
Physical inactivity	-0.0135	0.1103^d^	-0.2693^d^	0.2538^d^	0.0947^d^	-0.0487^b^	0.1186^d^	1.0000		
Depression	0.0529^c^	-0.0060	-0.0096	0.0564^c^	0.1768^d^	0.0943^d^	0.0913^d^	0.0727^d^	1.0000	
Anxiety	0.0537^d^	-0.0038	0.0462^b^	0.0154	0.1884^d^	0.0730^d^	0.0879^d^	-0.0187	0.3617^d^	1.0000

^a^*P *< 0.05; ^b^*P *< 0.01; ^c^*P *< 0.001; ^d^*P *< 0.0001.

**Table 5:** Stepped and full logistic regression models showing associations with CFS^a^

	Basic model **(*N *= 3,794; *R***^**2 **^**= 0.05)**			Basic model and social variables **(*N *= 3,741; *R***^**2 **^**= 0.14)**			Basic model and physical inactivity **(*N *= 3,792; *R***^**2 **^			Basic model and psychological variables **(*N *= 3,794; *R***^**2 **^			Full model ** (*N *= 3,739; *R***^**22 **^		
	
	OR	95% CI	*P *value	OR	95% CI	*P *value	OR	95% CI	*P *value	OR	95% CI	*P *value	OR	95% CI	*P *value
Ethnicity															
Irish	2.12	0.8 to 5.71	0.13	1.96	0.70 to 5.44	0.2	1.76	0.64 to 4.80	0.27	2.25	0.83 to 6.09	0.108	1.72	0.58 to 5.1	0.32
Black Caribbean	2.33	0.90 to 5.98	0.078	1.48	0.55 to 4.02	0.44	1.80	0.70 to 4.60	0.22	2.62	1.02 to 6.74	0.045	1.65	0.58 to 4.68	0.34
Bangladeshi	3.24	1.17 to 9.01	0.02	1.54	0.55 to 4.34	0.41	1.89	0.62 to 5.68	0.26	4.5	1.61 to 12.4	0.004	1.87	0.61 to 5.75	0.27
Indian	2.51	0.97 to 6.53	0.057	2.01	0.73 to 5.50	0.17	1.77	0.65 to 4.84	0.26	2.33	0.86 to 6.3	0.093	1.51	0.5 to 4.6	0.45
Pakistani	4.09	1.6 to 10.44	0.003	3.34	1.27 to 8.75	0.01	2.71	1.01 to 7.30	0.048	3.74	1.39 to 10.05	0.009	2.49	0.83 to 7.45	0.10
Sex	0.98	0.59 to 1.65	0.96	1.01	0.59 to 1.73	0.95	1.02	0.61 to 1.70	0.92	0.88	0.51 to 1.51	0.645	0.91	0.53 to 1.56	0.74
Age	1.02	1.01 to 1.03	0.002	1.03	1.02 to 1.05	<0.001	1.01	1.01 to 1.03	0.015	1.03	1.01 to 1.04	0.001	1.02	1.01 to 1.04	0.001
Education	0.95	0.52 to 1.74	0.87	0.85	0.45 to 1.59	0.61	0.98	0.53 to 1.82	0.96	0.91	0.48 to 1.75	0.795	0.85	0.44 to 1.66	0.65
Employment	1.18	0.66 to 2.12	0.56	1.20	0.66 to 2.18	0.55	1.07	0.59 to 1.93	0.82	0.90	0.49 to 1.68	0.760	0.86	0.44 to 1.68	0.67
Type of employment	1.27	0.74 to 2.17	0.38	1.24	0.71 to 2.17	0.44	1.22	0.71 to 2.09	0.46	1.26	0.71 to 2.24	0.41	1.18	0.65 to 2.14	0.57
Strain (score)				1.44	1.08 to 1.94	<0.0001							1.22	1.02 to 1.47	0.03
Discrimination (score)				1.45	1.08 to 1.95	0.01							1.28	0.92 to 1.78	0.15
Negative aspects of social support				2.12	1.37 to 3.28	0.001							2.0	1.25 to 3.2	0.004
Physical in activity (moderate/vigorous vs. inactive/light)							2.33	1.37 to 4.00	0.001				2.17	1.13 to 4.16	0.02
Depression score										1.59	1.27 to 1.99	0.001	1.49	1.17 to 1.89	0.001
Anxiety score										1.91	1.54 to 2.39	0.001	1.80	1.43 to 2.27	<0.001

^a^CFS, chronic fatigue syndrome; OR, odds ratio; 95% CI, 95% confidence interval. All variables were identified by likelihood ratio (LR) tests to contribute to the basic model, including age and socioeconomic status (SES) variables (education, none vs. some; employment of head of household, manual vs. nonmanual; employed, no vs. yes). Head of household and employment contributed significantly to the model when considered on their own, whereas education did not. However, when all three SES variables were considered together, they contributed significantly to the model. Sex did not contribute to models using the LR test and changed ORs only slightly. Age of migration and place of birth were tested after the demographic variables were entered, but did not contribute significantly to the model. They were also tested in the final model and made no difference in the results, so they were not retained. All analyses were weighted and adjusted for physical illnesses that might explain fatigue.

## Results

### Prevalence of CF and CFS

The weighted prevalence of CFS for the whole sample was 2.3% (108 of 4,273). The weighted prevalence of CF across the whole sample was 19.7% (881 of 4,276). CFS prevalence varied by ethnic group, age and educational level and was most prevalent among Pakistani people (Table 1). In contrast, the prevalence of CF varied only by age, sex and marital and employment status, but not by ethnic group (Table 1). The demographic factors associated with CF and CFS are similar (Table 1), but apart from the ethnic group differences, women and retired people have a higher risk of CF but not of CFS. Manual labour workers have a higher risk of CFS but not of CF. People over the age of 35 have a higher risk of CF and CFS in the univariate analyses (reported in Table 1). In the final model (Table 5), a 1-year increase in age is associated with a 2% (95% confidence interval (95% CI), 1% to 4%) higher risk of CFS. As CF did not vary by ethnic group, we did not undertake any further analyses of CF.

When considering univariate associations, chronic social strain, perceived discrimination, the negative aspects of social support, physical inactivity and anxiety and depression were each associated with a higher risk of CFS (Table 2).

Table 3 sets out ethnic variations in measures of social adversity, physical inactivity and anxiety and depression. The Bangladeshi group was more likely to score in the high range on social support (positive and negative) and on social strain. Perceived discrimination scores were highest among the Black Caribbean, Pakistani and Indian groups. Bangladeshi and Pakistani groups were least active as measured on the frequency and intensity of physical activity. Bangladeshi and Pakistani groups had the highest mean depression scores.

### Explaining ethnic variations in the prevalence of CFS

Before reporting regression models, Table 4 sets out the nonparametric correlations between demographic and risk factors entered into the final regression model. This shows highly significant correlations, which are addressed by including these variables in the regression models to show their independent effects.

In regression models, adding chronic strain, perceived discrimination and negative aspects of social support to the basic model led to some reduction in the risk among specific ethnic groups, thus partially explaining the higher prevalence of CFS in some ethnic groups (see Table 5). However, there was little reduction in risk for the Pakistani group (from an odds ratio (OR) of 4.1 (95% CI, 1.6 to 10.4) to an OR of 3.5 (95% CI, 1.4 to 9.1)). Adding physical inactivity reduced the risk of CFS to a nonsignificant level in all but the Pakistani group. Anxiety and depression alone did not account for ethnic variations in CFS prevalence.

In the full model, age, social strain, negative aspects of social support, physical inactivity and anxiety and depression were independent risk factors for CFS and together explained ethnic variations in CFS prevalence. The point estimates for the Pakistani group especially, and for the other ethnic groups, remained elevated (>1) but were not statistically significant. The full model explained 24% of the variance.

## Discussion

This is the first population study of both CF and CFS to include a large, ethnically diverse sample (six ethnic groups). The overall weighted prevalence of 2.3% for CFS is similar to the 2.6% found in the previous largest study of CFS in U.K. primary care [3]. Overall, the findings from this study suggest that most ethnic minority groups have a higher risk for CFS, but not for CF, when compared to White people. Although early studies indicated that CFS was uncommon among ethnic minorities [4,5], our study is consistent with research that shows that this may be related to the selection bias of clinic attenders rather than a lower prevalence of CFS [37].

Middle age is reported to be a high-risk period for developing CFS, and a higher risk is expected in those over 38 years of age [38]. Although many explanations exist for fatigue in older people, even after recovery from physical illness, the National Institute on Aging (in the United States) did not find a consistently higher risk among older adults or a rise in risk with age (http://www.nia.nih.gov/researchinformation/conferencesandmeetings/unexplainedfatigue.htm). Indeed, in contrast, a recent study validating a fatigue measure showed a lower fatigue score with increasing age [39]. We adjusted for physical illnesses that might explain fatigue symptoms, and therefore comorbid physical illnesses are unlikely to explain the findings of rising risk of CFS with age.

Overall, physically inactive people were twice as likely to have CFS compared with active individuals. However, physical inactivity may emerge following the onset of CFS as a way of avoiding fatigue; once physical inactivity emerges, deconditioning can occur and can further compound fatigue [25]. Prospective studies have suggested that fatigue is more commonly found in those who were physically active earlier in their lives [23]. In contrast to the longitudinal analysis of the 1946 birth cohort [24], we did not find a higher risk of CFS among the very active or the very inactive (data not shown). We could not identify a subgroup within our active group who might be irregularly involved in activity, as our estimates of activity were averages. Future research might also investigate whether increasing population levels of physical activity reduce the future risk of CFS or indeed increase it as suggested in one cohort study, as well as whether irregular patterns of activity rather than inactivity are significant predictors of fatigue [36].

Anxiety and depression did not alone explain the ethnic variations in CFS prevalence. The findings suggest that the risk of CFS overall is increased by 49% for each point on the depression score and by 80% for each point on the anxiety score. Although the survey was cross-sectional in nature and reverse causality may be an important explanation, previous research has shown that anxiety and depression can increase the risk of later CFS [23] and may share with CFS predisposing risk factors such as infectious illness [40,41].

CFS may reflect the influence of social status, power and exposure to adversity. Those with negative aspects of social support were twice as likely to have CFS, and those with social strain were 40% more likely to have CFS. Anthropological and biological critiques suggest that physiological symptoms, such as fatigue, can be an expression of social pressures related to minority status, including discrimination and social strain [42]. This has not been tested previously in epidemiological studies. This study offers some confirmation that chronic social strain and negative aspects of social support explain some of the higher risk of CFS among ethnic groups, but perceived discrimination was not statistically significantly associated with CFS in the final models. Ethnic group differences in the findings following adjustments are probably explained by each ethnic group's differing profile of risk factors; for example, exposure to discrimination and social strain, levels of physical inactivity and levels of anxiety and depression differ by ethnic group (Table 3). Our final models show independent effects of anxiety, depression, physical inactivity, social strain, negative aspects of social support and age in the whole population. Coping and help-seeking may also vary with culture and ethnic group [28]. We did not measure coping mechanisms or help-seeking for CF and CFS. As these are important in service-based studies that investigate access and recovery, they do not account for our population-based findings. Studies of recovery from CFS by ethnic group are needed to investigate whether different health beliefs and coping styles influence clinical outcomes. A qualitative component of the EMPIRIC study is investigating these aspects using secondary data analysis of qualitative data.

## Limitations

The study is the largest of ethnic groups in the United Kingdom, but even larger samples are needed to test for interactions between specific risk factors for specific ethnic groups. The classification of ethnic groups is undergoing constant revision. We were reliant on the categories used for the census in the United Kingdom, which were used in the HSE and EMPIRIC surveys. Ethnic minority status remains an important category by which inequalities are assessed. The treatment of anxiety and depression symptoms remains important, even if some consider that these are a consequence rather than a cause of CF and CFS. Studies of physical illness as aetiological factors in CFS are difficult to undertake, given the various exclusion criteria requiring detailed clinical information about the timing of fatigue and other illnesses. We were not able to address this question in this study. As we did not have biomarkers, this study was not able to test for other causes of fatigue, for example, we did not have measures of immune response or endocrine function.

The operational definition of CFS that we used in this study conforms to what has been called a 'CFS-like' illness in some published research; yet, even within the 'CFS-like' grouping, there are many variations in classification. Clinical studies and replication studies are necessary to test for risk factors amongst those developing CFS in accordance with full clinical diagnostic criteria. Similarly, we were not able to assess psychosomatic illnesses and their relationships with CFS. Clinical studies using independent and valid measures of psychosomatic illnesses in ethnic groups may be especially helpful, given that some ethnic groups are reported to have more psychosomatic illnesses.

Ethnic group-specific analysis might be helpful to establish risk factors for specific ethnic groups; however, although this study remains the largest population sample of ethnic groups in England, it is inadequately powered to assess the precise effects and interactions between the risk factors in each ethnic group separately.

## Conclusion

This study indicates that CFS is associated with social strain, negative aspects of social support, physical inactivity, anxiety and depression. These associations together explain the higher risks among some ethnic groups.

## Appendix: Fatigue questions

Have you noticed that you've been getting tired in the past month?

1. Yes

2. No

During the past month, have you felt you've been lacking in energy?

1. Yes

2. No

Do you know why you have been feeling tired or lacking in energy?

1. Yes

2. No

What is the main reason?

1. Problems with sleep

2. Medication

3. Physical illness

4. Working too hard (including housework, looking after baby)

5. Stress, worry or other psychological reason

6. Physical exercise

7. Other (specify)

In the past seven days, on how many days have you felt tired or lacking in energy?

1. 4 days or more

2. 1 to 3 days

3. None

## Competing interests

The authors declare that they have no competing interests.

## Authors' contributions

KSB conceived of the study, was the principal investigator, provided day-to-day management and drafted the manuscript. SD prepared and managed the data sets and carried out the statistical analyses. DA supervised statistical analyses and gave expert advice on nested models. JN and SW gave advice on all different phases of the study and gave particular help in forming the objectives of the study. PDW gave advice on the design and data analysis stages of the study, the definition of CFS and in shaping the conclusions of the study. All authors commented on and edited all drafts of the manuscript. All authors read and approved the final manuscript.

## Pre-publication history

The pre-publication history for this paper can be accessed here:

http://www.biomedcentral.com/1741-7015/9/26/prepub
